# Risk for Adolescent Substance Use Initiation: Associations with Large-Scale Brain Network Recruitment During Emotional Inhibitory Control

**DOI:** 10.3390/bs15101407

**Published:** 2025-10-16

**Authors:** Julia E. Cohen-Gilbert, Jennifer T. Sneider, Emily N. Oot, Anna M. Seraikas, Eleanor M. Schuttenberg, Sion K. Harris, Lisa D. Nickerson, Marisa M. Silveri

**Affiliations:** 1 Neurodevelopmental Laboratory on Addictions and Mental Health, McLean Hospital, Belmont, MA 02478, USA; jtsneider@mclean.harvard.edu (J.T.S.); eoot@bu.edu (E.N.O.); anna.seraikas@gmail.com (A.M.S.); eleanor.schuttenberg@maine.edu (E.M.S.); msilveri@mclean.harvard.edu (M.M.S.); 2 Department of Psychiatry, Harvard Medical School, Boston, MA 02115, USA; lisa_nickerson@hms.harvard.edu; 3 Applied Neuroimaging Statistics Research Lab, McLean Hospital, Belmont, MA 02478, USA; 4 Department of Psychiatry, Boston University School of Medicine, Boston, MA 02118, USA; 5 Boston Children’s Hospital, Boston, MA 02115, USA; sion.harris@childrens.harvard.edu; 6 Department of Pediatrics, Harvard Medical School, Boston, MA 02115, USA

**Keywords:** adolescence, substance use, inhibitory control, emotion, brain network, ICA, fMRI

## Abstract

As the brain continues to mature during adolescence, heightened impulsivity in emotional situations may increase the likelihood of initiating substance use. Functional magnetic resonance imaging (fMRI) was used to assess large-scale network activation during an emotional inhibitory control task (Go-NoGo). Participants were healthy, substance-naïve adolescents aged 13–14 years (*n* = 56, 31 females) who were then followed for 3 years with quarterly substance use evaluations. During follow-up, 24 participants initiated substance use, while 32 remained substance-naïve. Network activation strength was extracted for the Negative NoGo > Neutral NoGo contrast in the left and right lateral frontoparietal networks (lL-FPN, rL-FPN) and the dorsal attention network (DAN) for each participant. The impact of network activation strength on substance use initiation was analyzed via survival analysis (Cox regression). Reduced activation strength of the lL-FPN was associated with significantly higher hazard of initiation of substance use (*p* = 0.008). No significant effects were observed for rL-FPN or DAN. Diminished engagement of the lL-FPN during inhibitory control in negative versus neutral emotional contexts was associated with earlier substance use initiation. This pattern of network activation may represent a neurobiological marker of self-regulation vulnerability, highlighting a potential target for early identification and prevention strategies during adolescence.

## 1. Introduction

Early initiation of substance use is a well-established risk factor for the later development of alcohol and substance use disorders (Bravo et al., 2018). However, it is not yet well understood to what degree this increased risk is due to neurobiological changes driven by substance use and to what extent it reflects pre-existing traits and conditions. Adolescents who use substances experience a wide range of negative outcomes, including alcohol poisoning, overdoses, blackouts, injuries, automobile crashes, physical and sexual assaults, sexually transmitted infections, interpersonal problems, and poor academic performance (Hingson et al., 2017; Perkins, 2002). Nevertheless, total rates of alcohol and substance use disorders are continuing to escalate (SAMHSA, 2020). Importantly, longitudinal research demonstrates that adolescents who go on to initiate any form of substance use can be distinguished from non-initiating peers by pre-existing brain and behavioral differences, including heightened reward sensitivity, reduced inhibitory control, and altered brain network engagement during decision-making (Hulvershorn et al., 2015; Morales et al., 2018; Norman et al., 2011; Tervo-Clemmens et al., 2020). Characterizing these early differences offers an opportunity to pinpoint at-risk youth and to design more effective, targeted prevention efforts (Lees et al., 2020; McQuaid et al., 2022).

Adolescent initiation of substance use is shaped by social and environmental influences such as peer norms, parental monitoring, and community context but is also critically impacted by ongoing brain maturation (Silveri, 2012; Steinfeld & Torregrossa, 2023). Developmental models highlight that heightened reward responsiveness, alongside still-developing inhibitory control, creates a neurobiological window of vulnerability to substance use (Squeglia & Cservenka, 2017; Bava & Tapert, 2010). Adolescence is also marked by continued refinement of brain circuitry implicated in executive control (Galvan, 2021; Spear, 2000), producing a developmental gap in which altered functioning in emotion- and reward-driven systems occurs prior to full maturation of brain systems supporting cognitive regulation (Casey et al., 2016; Larsen & Luna, 2018; Luna et al., 2015). This imbalance can foster impulsivity and risk-taking behaviors, including substance use (Casey & Jones, 2010; Peeters et al., 2017; Steinberg, 2008), while heightened neurobiological plasticity further increases susceptibility to the adverse effects of substance use (Crews et al., 2016; Silveri et al., 2016; Spear, 2016).

Powerful emotions can increase impulsivity (Yong et al., 2010). Negative urgency, the tendency to act rashly during negative emotions, has been linked to problematic substance use (Berg et al., 2015; Dick et al., 2010) and use disorders (Fischer et al., 2004; Verdejo-Garcia et al., 2007). Impulsive errors on the Go-NoGo task, a measure of inhibitory control, correlate with higher negative urgency (Dick et al., 2010). Functional magnetic resonance imaging (fMRI) studies using Go-NoGo tasks have identified alterations in task-related brain activation associated with current and future substance use (Wetherill et al., 2013). Integrating negative emotional stimuli or distractors into a Go-NoGo protocol offers further insight into how negative emotions impact inhibitory control and brain activation. In healthy young adults, self-reported risk behavior tendencies were associated with greater orbital and ventromedial prefrontal activation during a Go-NoGo task with neutral or aversive image distractors (Brown et al., 2015). In contrast, recent binge drinking in young adults, during negative versus neutral distractor conditions of an emotional Go-NoGo task, was associated with decreased activation of brain regions implicated in inhibitory control: the anterior cingulate cortex (ACC), dorsolateral prefrontal cortex (DLPFC), and dorsomedial prefrontal cortex (DMPFC) (Cohen-Gilbert et al., 2017).

Large-scale networks are essential for understanding the neural basis of cognition and behavior. Functional connectivity closely aligns with brain architecture both at rest and during task performance, defining intrinsic large-scale brain networks (Cole et al., 2014; Laird et al., 2011; Nickerson, 2018; Smith et al., 2009). Among those studied in top-down cognitive control are the left and right lateral frontoparietal networks (lL-FPN and rL-FPN) and the dorsal attention network (DAN). The L-FPNs, comprising the lateral prefrontal cortex, posterior parietal lobe, intraparietal sulcus, and ventral inferior temporal lobe, both support cognitive flexibility and attention switching. While some previous work has treated right and left L-FPN as a single network (Yeo et al., 2011), sometimes termed the central executive network, mounting evidence shows that it reproducibly separates into lateralized sub-networks (Dosenbach et al., 2008; Smith et al., 2009; Uddin et al., 2019) with closely related functions but dissociable patterns of connectivity that follow distinct developmental trajectories during adolescence (Lund et al., 2022). The DAN, encompassing DLPFC, frontal eye fields, superior parietal lobe, and middle temporal motion complex, supports top-down control of externally directed attention, particularly the orienting and shifting of focus toward relevant stimuli (Corbetta et al., 2008; Corbetta & Shulman, 2002; Uddin et al., 2019).

A prospective, preliminary examination of young adult binge drinkers found that the maximum number of drinks consumed per occasion was associated with reduced average activation of a frontoparietal network and further reported that an escalation in the maximum number of drinks during a 12-month follow-up was associated with a greater magnitude of difference in engagement of this network during successful inhibition versus error trials in a Go-NoGo task and with higher self-reported impulsivity (Worhunsky et al., 2016). In older adolescents, lower activation of the DAN during negative versus neutral conditions of an emotional Go-NoGo task correlated with higher Alcohol Use Disorders Identification Test (AUDIT) scores and other substance use measures (Cohen-Gilbert et al., 2022). This study also reported a positive association between rL-FPN activation and substance use one year later. Although evidence for the role of large-scale networks in substance use is growing, most work has focused on resting state network connectivity (Wilcox et al., 2019).

This study aimed to identify neurobiological markers present before substance use initiation that may confer risk for earlier use onset, specifically, the impact of negative emotions on the activity of executive and attentional control networks during response inhibition. To this end, this study examines DAN and L-FPN engagement during the performance of negative versus neutral distractor condition of an emotional Go-NoGo task. Using multivariate methods (Nickerson, 2018), task-related network loadings were extracted to quantify activation strength of DAN, rL-FPN and lL-FPN in each individual, while accounting for overlapping networks. Survival analysis (Cox regression) was then used to examine whether network activation strengths predicted time to substance use initiation in healthy, substance-naïve adolescents. We hypothesized that failure to recruit executive networks during emotional distraction (i.e., less recruitment of rL-FPN, lL-FPN and DAN during negative versus neutral trials) would be associated with earlier substance use initiation and poorer task performance, particularly during negative emotional distraction. Characterization of network engagement during emotional response inhibition may help identify brain correlates of self-regulation deficits that predict vulnerability to substance use during adolescence.

## 2. Method

### 2.1. Participants

Participants included in these analyses were 56 healthy adolescents (aged 13–14 years during the baseline assessment, 31 female) with up to 4.5 years of follow-up data available. All participants were substance-naïve at the time of baseline scanning, and 24 individuals initiated use during the follow-up period. Mean age of initiation of substance use among initiators was 15.3 ± 1.2 years old (range 13.5 to 18.0 years). The majority of participants who initiated substance use during the study period initiated multiple substances (75%). The average number of substances used was 2.3 ± 1.0. Substances used in the initiator group included alcohol (83%), cannabis (71%), cigarettes (33%), any vaping (33%), inhalants (8%), and prescription stimulants (8%). Demographic and IQ data are summarized in Table 1. All participants in this sample reported gender identities aligned with sex at birth. Participants were screened for neurological disorders, current psychotropic medication use, head trauma with loss of consciousness, and MRI contraindications, such as claustrophobia or metal in the body. All participants completed urine screening before baseline scanning to rule out any current psychoactive substance use (Clarity Diagnostics Drugs of Abuse Panel, Boca Raton, FL, USA) and pregnancy in females (QuPID One-Step Pregnancy, Stanbio Laboratory, Inc., San Antonio, TX, USA). At baseline, participants had no lifetime psychiatric diagnoses and were further screened for psychological disorders using the Mini International Neuropsychiatric Interview for Children and Adolescents (MINI-KID, Sheehan et al., 2010). IQ was assessed via the Wechsler Abbreviated Scale of Intelligence (WASI, two-subscale IQ) (Wechsler, 1999). Pubertal status was measured via self-report using the Petersen scale (Petersen et al., 1988). Parents or legal guardians of all participants provided written informed consent prior to participation, and all participants provided assent. The study was conducted in accordance with the Declaration of Helsinki and approved by the Institutional Review Board of McLean Hospital, part of Mass General Brigham (Protocol Number: 2013P002420, Date of Approval: 14 August 2018). Participants received monetary compensation for study participation.

### 2.2. Substance Use Initiation

Substance use surveys were self-administered using REDCap software hosted at Partners HealthCare (now Mass General Brigham), which was running versions 8.9 to 9.1 (Harris et al., 2009) and included items from the CRAFFT screening tool (Knight et al., 2002) and the Texas Christian University Drug Screen 5 (TCU DS 5; Wiese et al., 2022), first during the baseline visit and then at regular quarterly intervals during the follow-up period (up to 4.5 years). Adolescents reported past three-month use of alcohol (excluding religious use, a single glass permitted by parents on special occasions, or a few sips), cannabis, nicotine (cigarettes, other tobacco products, vaping), inhalants/solvents, prescription drugs without a prescription (opioid pain relievers, stimulants), and other illicit substances (e.g., heroin, cocaine). Response options ranged from Never to Daily. Age of substance use initiation was defined as the first incidence (age) of any substance use. Age of substance use initiation was further queried via a self-administered REDCap exit survey at the end of the study participation period. While quarterly reports served as the primary source of initiation data, exit survey data were used to confirm reported initiation dates and fill data gaps.

**Figure 1:**
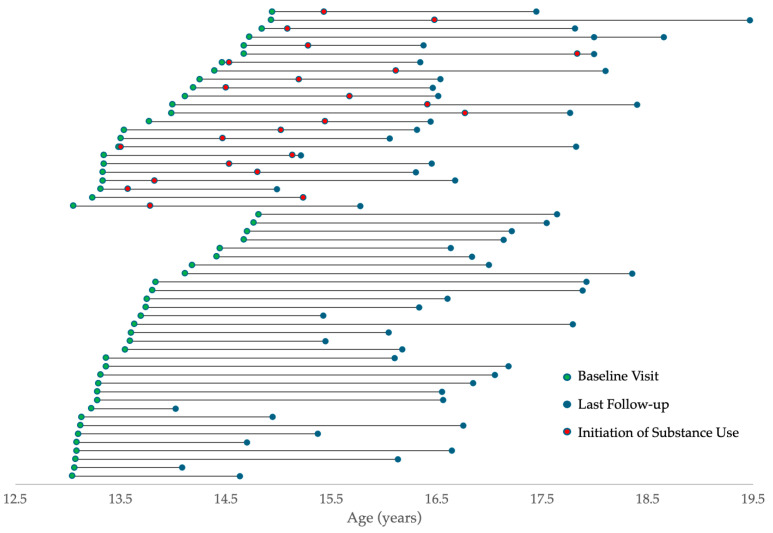
Timing of baseline visits and final follow-up data for all participants and age of first substance use for those who reported initiating during follow-up (top group).

Participants included in this sample represent a subset of participants from a larger longitudinal MRI project (*n* = 81). Data included in the analyses were from participants who met the eligibility criteria and had high-quality structural and functional MRI data, with a final sample size of *n* = 56 with 24 participants reporting substance use during follow-up. Duration of follow-up was not significantly different between initiators and non-initiators, F(1,54) = 0.345, *p* = 0.559. Baseline ages, ages of final follow-up data and ages of substance use initiation are depicted in Figure 1. Given the wide range of follow-up durations, no direct contrasts of initiators versus non-initiator groups were conducted, given that participants lost to follow-up earlier may have gone on the initiate substance use. For participants who remained in the study until age 16 (*n* = 48), group comparisons are provided in the Supplementary Materials (Tables S1 and S2), showing no group differences in demographic variables or task performance variables between participants who initiated substance use prior to versus after age 16.

### 2.3. Emotional Go-NoGo Task

FMRI data were acquired during an emotional Go-NoGo task that required participants to ignore background images with positive, negative, or neutral emotional valence (see Cohen-Gilbert et al., 2017, for a full description). As in the prior study, in the Go-NoGo task, letters were presented sequentially within a small box at the center of the background image. Background images were selected based on valence ratings from the International Affective Picture System (IAPS) (Lang et al., 2008) to represent positive, negative, or neutral conditions. Scrambled versions of these images, with no discernible image content, served as non-emotional control stimuli. The task consisted of 480 trials, evenly split between background types (120 each). Each background image was presented once during the task. Participants were instructed to respond (button press) as quickly as possible to every letter stimulus, except for the letter ‘X’. Participant responses were recorded using a fiber-optic response pad (FORP). Xs appeared in 25% of the trials, such that participants acquired a prepotent tendency to press, and NoGo trials required active inhibition of a response. The task, presented using E-prime 2, was synched to the scanner via RF pulse. The paradigm used a rapid event-related design, with each 1500 ms trial consisting of 500 ms of fixation, followed by 350 ms of the background image presented alone, and then 650 ms in which the letter cue and background image were both visible. This rapid stimulus presentation creates high levels of inhibitory demand and prevents ceiling effects in NoGo trial performance in this healthy sample. Target NoGo trials were distributed strategically within the Go trial stream to create jitter. Go trials were treated as an implicit baseline and, therefore, not modeled (Garavan et al., 2002). The task was presented in three runs (160 trials/run). Task performance measures included accuracy on NoGo trials, accuracy on Go trials, and reaction times on correct Go trials.

### 2.4. Magnetic Resonance Imaging

All MRI data were collected using a 3 Tesla Siemens TIM Trio scanner (Erlangen, Germany) with a 32-channel head coil. High-resolution structural images were acquired using a T1-weighted multi-echo Multiplanar Rapidly Acquired Gradient-Echo (ME-MPRAGE) 3D sequence in 4 echoes (TE = 1.64/3.5/5.36/7.22 ms, TR = 2.1 s, TI = 1.1 s, FA = 12°, 176 slices, 1 × 1 × 1.3 mm voxel, acquisition time = 5 min) and were used to register functional images to standard space. FMRI data were collected in three runs (5:13 min/run) using whole-brain multiband gradient-echo echo-planar imaging (EPI) with BOLD contrast. Images were acquired in 54 oblique, interleaved slices (TR/TE/FA = 750 ms/30 ms/52°, FOV = 220, voxel size: 2.8 mm × 2.8 mm × 2.8 mm, multiband = 6, GRAPPA = 2). A field map (TR = 1000, TE = 10/12.46 ms, FA = 90°, 2:44 min) was acquired at the same resolution and slice locations to provide offline correction of field inhomogeneities.

### 2.5. FMRI Data Processing

#### 2.5.1. Data Preprocessing

FMRIB Software Library (FSL) software v6.0.11 (Smith et al., 2004) was used to preprocess raw data and for statistical analyses. Preprocessing steps included motion correction, slice-timing correction, non-brain removal, spatial smoothing (FWHM 6 mm Gaussian kernel), and grand-mean intensity normalization of the 4D dataset by a single multiplicative factor. Runs began with a 30 s rest block (40 volumes) before task onset, which was removed prior to analysis; thus, no additional volumes were removed to allow signal equilibration. Within the selected sample, motion did not exceed one voxel. ICA-AROMA, an independent component analysis-based denoising tool, was applied to further correct for motion and physiological artifacts (Pruim et al., 2015). Following ICA-based denoising, data underwent temporal filtering using a Gaussian-weighted least-squares straight line fit with a highpass cutoff of 100 s and field map-based distortion correction. FMRI data were registered to MNI152 standard space (Mazziotta et al., 2001a, 2001b), using FNIRT, to first register data to each individual’s high-resolution structural image using boundary-based registration (BBR), then apply the registration information derived from registering the high-resolution structural image to MNI152 standard space.

#### 2.5.2. fMRI Statistical Modeling

A voxel-wise general linear model (GLM) with all NoGo trials within the four background conditions (positive, negative, neutral, scrambled), each modeled as separate regressors (30 trials per condition), convolved with a gamma hemodynamic response function, was implemented for each run. Contrasts of parameter estimates (COPE) maps were calculated for the contrast of interest: Negative NoGo v. Neutral NoGo. COPEs from the three task runs were combined using a second-level fixed effects GLM to create an average COPE map for each participant. Average COPE maps were then used for further statistical analyses.

#### 2.5.3. Extraction of Network Activation Weights

**Figure 2:**
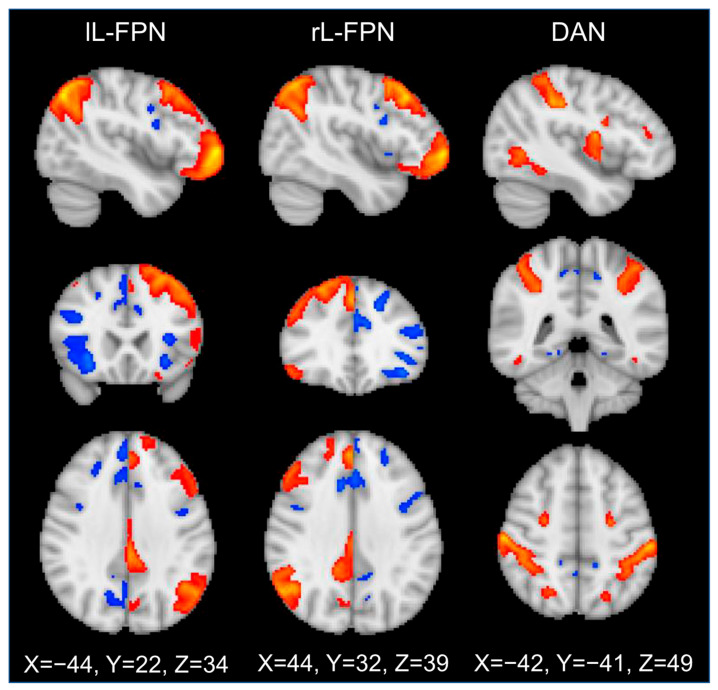
Template networks of interest, extracted from Human Connectome Project data (https://www.humanconnectome.org/study/hcp-young-adult, *n* = 1003, dimensionality = 50 components, HCP 1200 release date: 21 July 2017) for the left lateral frontoparietal network (lL-FPN), the right lateral frontoparietal control network (rL-FPN), and the dorsal attention network (DAN). Orange network areas activation is positively correlated over time, while blue regions show a significant negative temporal correlation with orange areas.

Task-related activation of key brain networks was extracted via a multivariate spatial regression approach (see Nickerson, 2018). Thus, participant-level average COPE maps were concatenated to generate a single 4D data file for the Negative NoGo > Neutral NoGo contrast. Open access network template spatial maps, derived from Human Connectome Project data (Smith et al., 2015) using group independent component analysis (https://www.humanconnectome.org/study/hcp-young-adult, *n* = 1003, dimensionality = 50 components, HCP 1200 release date: 21 July 2017), were then spatially regressed against the concatenated participant-level brain activation maps to estimate the impact of negative emotional stimuli during response inhibition on the strength of activation of each associated network. Task-related network loadings thus reflected the activation strength of individual networks for each participant, taking into account any spatial overlap among networks. The advantage of this approach is that the average activation strength of a particular network can be disentangled from other networks. Thus, using multivariate spatial regression against network templates allows activity from individual networks to be disentangled despite spatial overlap. Networks of interest were three networks previously implicated in top-down regulation of attention and cognition: left and right L-FPN (lL-FPN; rL-FPN) and DAN. Templates used for these three networks of interest are shown in Figure 2. The L-FPN was examined bilaterally because it reproducibly separates into two lateralized sub-networks (Laird et al., 2011; Nickerson, 2018; Smith et al., 2009). The DAN was examined as a single bilateral network (Corbetta et al., 2008; Dosenbach et al., 2008). HCP templates for the a priori networks were selected because they were derived from a large sample of healthy young people (*n* = 1003; four runs of 15 min of high-quality data per subject). These templates comprised network template spatial maps corresponding to highly reproducible task networks (Laird et al., 2011; Nickerson, 2018; Smith et al., 2009).

### 2.6. Statistical Analysis

For individuals who initiated substance use during follow-up (*n* = 24), time to initiation was computed by subtracting age at baseline scan from age of first reported use. For those who did not initiate (censored, *n* = 32), the duration of follow-up was computed by subtracting age at baseline scan from the age at which the last report of non-use was acquired. These values were integrated into a single outcome variable for survival analysis. A Cox proportional hazards regression model was used to examine the association between predictors of interest (task performance, network activation) and time to substance use initiation from age at baseline. All models included the full sample (*n* = 56).

As the likelihood of substance use initiation increases with age, age at baseline was also included as a covariate in all analyses. Recent epidemiological studies have reported few sex differences in adolescent substance use initiation (e.g., Bhatia et al., 2023; Kuhn, 2015), which are not uniform across substances, ages, or stages of use (Goodwin et al., 2022). However, we included sex as a covariate given robust evidence for sex differences in adolescent brain development that are directly relevant to substance use vulnerability (Kaczkurkin et al., 2019; Lenroot & Giedd, 2010; Vijayakumar et al., 2018).

#### 2.6.1. Behavioral Measures

Three repeated measures ANOVAs were performed to examine the impact of the emotional backgrounds (negative vs. neutral) on task performance (accuracy on NoGo trials, accuracy on Go trials, and reaction time on correct Go trials). Sex was included as a between-subject predictor and age was included as a covariate.

Six Cox regressions were used to examine whether task performance for each background condition was associated with relative risk for substance use initiation. A Bonferroni correction was applied to account for multiple analyses (α = 0.008).

#### 2.6.2. Network Activation

Three Cox regressions were used to examine the impact of activation strength in the three networks of interest (lL-FPN, rL-FPN, and DAN) on time to substance use initiation. A Bonferroni correction was applied to account for multiple analyses (α = 0.016).

Three multiple linear regressions were performed to examine associations between network activation strength and inhibitory control (NoGo trial accuracy), with activation strength of the three networks of interest as independent variables (IVs) and age and sex as covariates. NoGo trial accuracy (dependent variable, DV) was examined separately for negative and neutral trials. To parallel the Negative NoGo > Neutral NoGo contrast used to extract network activation strength, the relative impact of the two background types on performance (negative–neutral) was also examined as a DV. A Bonferroni correction was applied to account for multiple analyses (α = 0.016).

Three additional multiple linear regressions were performed, with activation strength of the three networks of interest as IVs, to examine associations between activation strength and processing speed in negative and neutral background conditions. Reaction time on correct Go trials (DV) was analyzed separately for negative and neutral trials and the difference in reaction times between the two conditions (negative–neutral). A Bonferroni correction was applied to account for multiple analyses (α = 0.016).

## 3. Results

### 3.1. Behavioral Measures

Task performance for the total sample is summarized in Table 2.

No significant impact of trial background on task performance was found in this sample: NoGo accuracy, F(1,53) = 0.01, *p* = 0.922; Go accuracy, F(1,53) = 0.83, *p* = 0.366; Go RT, F(1,53) = 1.36, *p* = 0.248. Variation in task performance measures was not significantly associated with hazard for substance use initiation (see Table 3).

### 3.2. Network Activation and Substance Use Initiation

**Figure 3:**
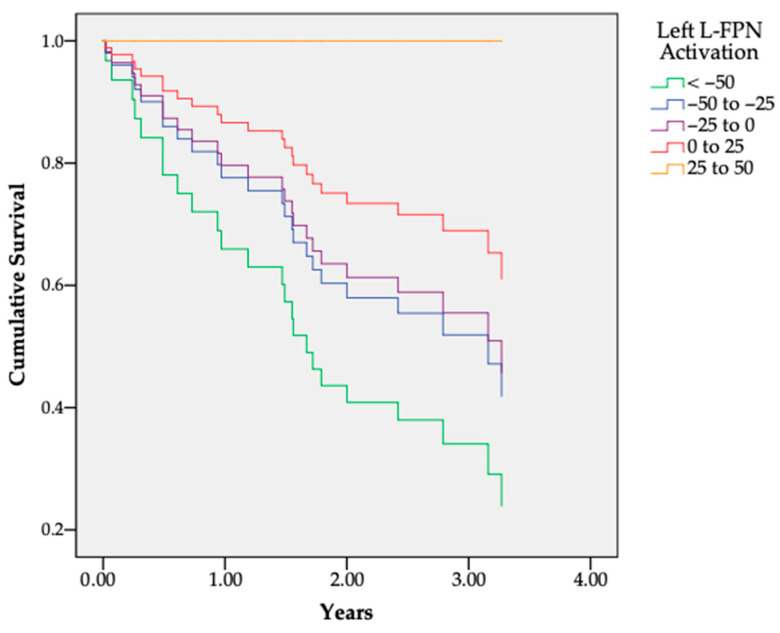
Sample survival curves for binned values left L-FPN activation on the Negative NoGo > Neutral NoGo contrast. Lower activation of this network was significantly associated with increased hazard for substance use initiation.

Activation strength of lL-FPN on the Negative NoGo > Neutral NoGo contrast was significantly associated with a reduced hazard of substance use initiation (longer time to event): Beta = −0.013, SE = 0.005, Wald = 6.94, df = 1, *p* = 0.008, Hazard Ratio = 0.988, 95% CI = 0.978, 0.997. Thus, conversely, increased suppression of this network during inhibitory control in the presence of negative versus neutral distractors was associated with a greater instantaneous risk of substance use initiation (lower time to event). Survival curves for binned lL-FPN are shown in Figure 3.

Activation strengths of rL-FPN (Beta = −0.006, SE = 0.006, Wald = 0.830, df = 1, *p* = 0.362, Hazard Ratio = 0.994, 95% CI = 0.982, 1.01) and DAN (Beta = −0.012, SE = 0.009, Wald = 1.86, df = 1, *p* = 0.173, Hazard Ratio = 0.988, 95% CI = 0.971, 1.01) were not significantly associated with substance use initiation, nor were any significant effects of sex or baseline age on use initiation observed in these models.

Multiple linear regressions revealed no significant associations between network activation and task performance measures, nor were any significant effects of age or sex on task performance observed in these models (see Table 4 and Table 5).

## 4. Discussion

This study examined activation of large-scale regulatory brain networks during inhibitory control efforts in the presence of emotionally negative versus neutral distractors in adolescents. Findings suggest that failure to adequately recruit regulatory circuitry under negative emotion conditions may represent a risk factor for earlier initiation of substance use. Specifically, less recruitment of the lL-FPN, a network implicated in executive function, was associated with increased risk of earlier substance use initiation. These findings suggest that adolescents for whom negative emotional information lessens engagement of executive control circuitry may be more likely to engage in early substance use.

The three large-scale brain networks examined—lL-FPN, rL-FPN, and DAN—play interconnected roles in filtering sensory information, aligning it with internal goals, and guiding behavioral responses (Laird et al., 2011). Altered functioning of these networks has been linked to substance use disorders (Wilcox et al., 2019; Zhu et al., 2017) and risky drinking (Sousa et al., 2019; Worhunsky et al., 2016). Furthermore, problematic alcohol use in first-year college students was found to be associated with activation of DAN during a comparable Go-NoGo task, both at baseline and at a one-year follow-up, while rL-FPN was associated more broadly with substance use at follow-up (Cohen-Gilbert et al., 2022). By contrast, in the current study, lL-FPN network activation strength showed the most robust relationship with initiation of substance use. These distinct, though interrelated, results may stem from developmental changes in network functioning and connectivity which occur during adolescence (Lund et al., 2022). Furthermore, the current findings highlight brain differences that precede and may increase risk for earlier substance use initiation across multiple substances, while findings in the college-aged group likely reflect both the impact of past and current alcohol use as well as risk factors for continued use. While it is tempting to theorize about the cognitive implications of the observed associations with distinct regulatory brain networks, any interpretations must remain highly speculative in the absence of direct associations between network activation and task performance.

Similarly to the prior studies in older adolescents (Cohen-Gilbert et al., 2017; Cohen-Gilbert et al., 2022), the current study found worse overall task performance and reduced impact of negative distractors on task performance compared to similarly aged adolescents performing an equivalent emotional Go-NoGo task outside the scanner (Cohen-Gilbert & Thomas, 2013).

Sex did not emerge as a significant predictor in any of the analyses, but the sample size limited examination of moderating effects. Alcohol misuse is higher in males than females, though this gap has narrowed over the past decade, particularly among youth (Dir et al., 2017), and prior research suggests that males, on average, show greater impulsivity and risk-taking. However, female adolescents are more likely to drink or use drugs in response to stress or negative emotions (Cross et al., 2011), making emotion-related impulsivity a vital factor in problematic substance use in this population (McHugh et al., 2018). Indeed, affective reactivity is associated with heavy drinking in female but not male young adults (Pedrelli et al., 2018). Considering these sex differences, network activation patterns related to emotional impulsivity and alcohol misuse may vary by sex and warrant further investigation.

Several limitations should be noted. First, correct and incorrect NoGo trials were combined within regressors due to the relative infrequency of NoGo events and variability in accuracy, which could confound activation estimates. However, performance did not differ significantly between negative and neutral NoGo conditions, supporting the interpretation that contrasts primarily reflect emotional influences on inhibitory control rather than success versus failure. Second, the sample size limited statistical power to detect effects, including moderating effects (e.g., sex, impulsivity), and future work with larger samples is needed to address these factors. Participant-related factors also warrant consideration. Substance use initiation was classified dichotomously (yes/no), an approach that maximized power for survival analyses but did not capture variation in timing, frequency, or type of substance use. The sample was further restricted to psychiatrically healthy, substance-naïve adolescents aged 13–14, limiting generalizability to higher-risk youth with earlier initiation or co-occurring psychopathology. In addition, network activation was assessed only at baseline; longitudinal imaging will be necessary for determining whether developmental changes in activation trajectories predict subsequent substance use outcomes.

## 5. Conclusions

Overall, these findings demonstrate the utility of large-scale brain network activation for probing functional brain responses during an emotionally challenging inhibitory control task. Network-level analyses offer a valuable complement to more fine-grained methods by allowing theory-driven tests of how executive and attentional systems function under conditions relevant to risk. In this study, weaker engagement of the lL-FPN during negative emotional contexts was associated with earlier substance use initiation, pointing to large-scale activation patterns as potential early markers of vulnerability. Considering adolescent risk through the lens of network-level brain function deepens our understanding of the neural basis of self-regulation and points to potential markers for early identification and prevention of substance use.

## Figures and Tables

**Table 1:** Participant Demographics and IQ.

Age (years)	13.8 ± 0.6
Female/Male	31/25
Education (years)	7.5 ± 0.7
SES ^a^	52.1 ± 8.3
Handedness	55R, 1L
Ethnicity ^b^	98% Non-Hispanic 2% Hispanic
Race ^c^	80% Caucasian 5% Asian 15% Multi-racial
WASI IQ estimate (2-subtest)	115.2 ± 10.5
Puberty ^d^	2.8 ± 0.7

Data represent means ± standard deviation. Abbreviations: ^a^ SES, Socioeconomic status (Hollingshead, 1975); WASI, Wechsler Abbreviated Scale of Intelligence; ^b^ Ethnicity: Hispanic vs. Non-Hispanic; ^c^ Race: “Multi-racial category” included the following designations: Asian/Caucasian; African American/Caucasian, American Indian or Native Alaskan/Caucasian; ^d^ Pubertal Development Score (Petersen et al., 1988).

**Table 2:** Task performance. Mean (standard deviation).

Measure	Negative	Neutral
NoGo accuracy (%)	53.10 (16.74)	57.38 (18.87)
Go accuracy (%)	97.46 (3.39)	98.33 (2.90)
Go reaction time (ms)	376.51 (47.52)	361.77 (39.42)

**Table 3:** Survival analysis of task performance and relative risk of for earlier substance use initiation.

	Beta	SE	Wald	df	*p*	Hazard Ratio	95% CI	
Negative NoGo Accuracy	−0.006	0.012	0.219	1	0.64	0.994	0.971	1.02
Negative Go Accuracy	0.066	0.089	0.541	1	0.462	1.068	0.897	1.272
Negative Go RT	−0.003	0.005	0.359	1	0.549	0.997	0.988	1.01
Neutral NoGo Accuracy	−0.009	0.011	0.664	1	0.415	0.991	0.969	1.01
Neutral Go Accuracy	−0.013	0.098	0.018	1	0.892	0.987	0.815	1.195
Neutral Go RT	−0.004	0.005	0.432	1	0.511	0.996	0.986	1.01

**Table 4:** Network activation on Negative NoGo > Neutral NoGo contrast: associations with NoGo trial accuracy.

	Negative		Neutral		Negative-Neutral	
Network	Standard β	*p*	Standard β	*p*	Standard β	*p*
left L-FPN	−0.121	0.378	−0.188	0.170	0.130	0.352
right L-FPN	0.028	0.846	0.054	0.713	−0.046	0.756
DAN	−0.116	0.401	−0.012	0.991	−0.163	0.245

**Table 5:** Network activation on Negative NoGo > Neutral NoGo contrast: associations with Go trial reaction times.

	Negative		Neutral		Negative-Neutral	
Network	Standard β	*p*	Standard β	*p*	Standard β	*p*
left L-FPN	−0.039	0.774	−0.016	0.908	−0.071	0.601
right L-FPN	0.188	0.190	0.174	0.232	0.120	0.403
DAN	>0.001	0.999	0.032	0.819	−0.073	0.592

## Data Availability

The data that support the findings of this study are not publicly available due to privacy and ethical restrictions. Requests for access to de-identified data may be considered by the corresponding author on reasonable request and in accordance with institutional policies.

## Supplementary Materials

The following supporting information can be downloaded at: https://www.mdpi.com/article/10.3390/bs15101407/s1, Table S1. Participant Demographics and IQ for a subsample with data up to age 16 years. Initiators here are defined as those who initiated substance use prior to 16 years while non-initiators did not; Table S2. Task performance. Mean (standard deviation) for a subsample with data up to age 16 years. Initiators here are defined as those who initiated substance use prior to 16 years while non-initiators did not.
