# Supplementary material for: Risk for Adolescent Substance Use Initiation: Associations with Large-Scale Brain Network Recruitment During Emotional Inhibitory Control
Source: Behav Sci (Basel). 2025 Oct 16;15(10):1407. doi: 10.3390/bs15101407 (PMC12561812; doi:10.3390/bs15101407)
Supplement: Supplementary file 1 [file behavsci-15-01407-s001.zip › behavsci-3861863-supplementary.pdf]

Supplementary materials

Table S1. Participant Demographics and IQ for a subsample with data up to age 16 years. Initiators here are defined as those who initiated substance use prior to 16 years while non-initiators did not.

	**Initiators (n = 18)**	**Non-Initiators (n = 30)**	**Total Sample (n= 48)**
Age (years)	13.8 ± 0.6	13.9 ± 0.6	13.9 ± 0.6
Female/Male	10/8	16/14	26/22
Education (years)	7.5 ± 0.7	7.7 ± 0.7	7.6 ± 0.7
SES[a]	53.9 ± 7.1	50.9 ± 8.5	52.1 ± 8.1
Handedness	18R	29R, 1L	47R, 1L
Ethnicity[b]	95%	100%	98% Non-Hispanic
	5%	0%	2% Hispanic
Race[c]	83%	80%	81% Caucasian
	0%	10%	6% Asian
	17%	10%	13% Multi-racial
WASI IQ estimate (2-subtest)	113.3 ± 12.1	116.37 ± 10.2	115.2 ± 10.9
Puberty[d]	2.7 ± 0.7	2.9 ± 0.6	2.8 ± 0.7

Data represent means ± standard deviation. Abbreviations: [a]SES, Socioeconomic status (Hollingshead, 1975); WASI, Wechsler Abbreviated Scale of Intelligence; [b]Ethnicity: Hispanic vs. Non-Hispanic; [c]Race: "Multi-racial category" included the following designations: Asian/Caucasian; African American/Caucasian, American Indian or Native Alaskan/ Caucasian; [d]Pubertal Development Score (Petersen et al., 1988). No significant differences were identified between initiator and non-initiator groups for sex, handedness, ethnicity, race, age at baseline, IQ, SES score, years of education, or pubertal score.

Table S2. Task performance. Mean (standard deviation) for a subsample with data up to age 16 years. Initiators here are defined as those who initiated substance use prior to 16 years while non-initiators did not.

	Negative		Neutral	
Measure	Initiators	Non-Initiators	Initiators	Non-Initiators
NoGo accuracy (%)	51.85 (16.10)	53.67 (18.08)	55.00 (19.07)	60.11 (19.80)
Go accuracy (%)	97.96 (2.26)	97.45 (3.60)	98.39 (2.53)	98.81 (1.93)
Go reaction time (msec)	371.46 (44.42)	373.77 (42.86)	357.28 (31.67)	358.37 (38.60)

No significant main or interaction effects of group (Initiator vs. Non-initiator), trial background or sex were observed on the three task performance measures.
